# Transcriptomic advances in studies of muscle stem cell aging: From bulk to single-cell and beyond

**DOI:** 10.1038/s41422-026-01240-w

**Published:** 2026-03-25

**Authors:** Soochi Kim, Seung Pil Pack, Thomas A. Rando

**Affiliations:** 1 Department of Biotechnology and Bioinformatics, Korea University, Sejong, Republic of Korea; 2 Broad Stem Cell Research Center, University of California, Los Angeles, Los Angeles, CA USA; 3 Department of Neurology, University of California, Los Angeles, Los Angeles, CA USA

**Keywords:** Ageing, Muscle stem cells

## Abstract

Advances in transcriptomic technologies have progressively transformed the questions we can ask and answer about muscle stem cells (MuSCs) during aging. Early microarray and bulk RNA sequencing studies established foundational population-level signatures of aged MuSCs, including attenuation of myogenic and metabolic programs as well as induction of inflammatory and stress-associated transcription. However, these averaged readouts obscured cell-to-cell variability and rare functional states. The transition to single-cell and single-nucleus RNA sequencing marked a turning point by resolving MuSC heterogeneity and revealing that MuSC aging is not purely stochastic. Instead, aged MuSC pools show reproducible changes in state composition, delayed or altered myogenic lineage progression, and selective vulnerability of specific functional subsets. Emerging spatial transcriptomic approaches, although still limited by sensitivity and cell-type discrimination in muscle, are beginning to place these MuSC states into their native tissue context, directly linking transcriptional states, niche organization, and age-associated remodeling. In parallel, integrative multi-omic designs that pair transcriptomics with chromatin accessibility and metabolic measurements have strengthened mechanistic connections among age-associated gene programs, epigenetic remodeling, and metabolic state shifts. Finally, computational frameworks — including trajectory inference, dynamic modeling, and machine learning — are increasingly applied to high-dimensional transcriptomic data to predict aging trajectories and identify candidate rejuvenation targets. In this Perspective, we trace the evolution of transcriptomic technologies through the lens of MuSC aging and highlight how increasing resolution has reframed core models of MuSC decline and plasticity.

## Introduction

Skeletal muscle regeneration depends on the activity of muscle stem cells (MuSCs), also known as satellite cells, which reside in a quiescent state beneath the basal lamina of muscle fibers.^1–3^ Upon injury or stress, MuSCs activate, proliferate, and differentiate to repair damaged tissue while maintaining the stem cell pool through self-renewal.^4,5^ With aging, however, this tightly regulated balance becomes disrupted. Aged MuSCs exhibit reduced regenerative capacity, increased susceptibility to senescence, and altered responses to environmental cues.^6–8^

Understanding the molecular mechanisms that underlie MuSC aging has long been a central challenge in regenerative biology. MuSCs have served as a model system for the study of stem cell aging,^9,10^ in part because they can be unequivocally identified in situ and prospectively isolated based on the expression of proteins that distinguish them from all other cell types in muscle tissue.^11–13^ Over the past decades, molecular characterization has followed functional characterization, with transcriptomic analyses leading the way in unbiased screening, as they have for most cells and tissues.

**Fig. 1:**
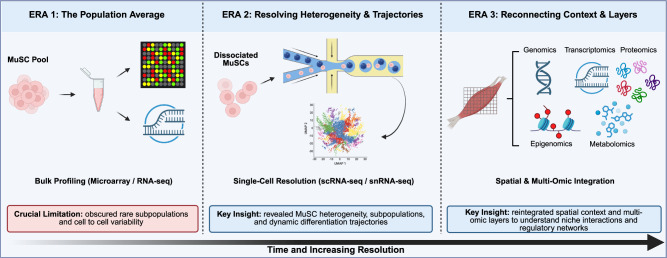
The evolution of transcriptomic resolution reveals MuSC heterogeneity and niche context during aging. This timeline illustrates three eras of technological advancement (bottom arrow) that have progressively increased the resolution of research on aged muscle stem cells (MuSCs). ERA 1 (left): Early bulk profiling methods (microarray, bulk RNA-seq) averaged transcriptional signals across heterogeneous populations. ERA 2 (middle): The advent of single-cell resolution (scRNA-seq, snRNA-seq) enabled dissection of dissociated MuSCs. Key insight: these advances revealed cellular heterogeneity within the MuSC pool, identified distinct subpopulations, and enabled reconstruction of dynamic differentiation trajectories. ERA 3 (right): Emerging spatial technologies preserve native tissue architecture, while multi-omic integration captures diverse molecular layers. Key insight: by integrating spatial context with multiple omics layers (e.g., genomics, transcriptomics, proteomics, epigenomics, and metabolomics), these approaches enable a deeper understanding of MuSC–niche interactions and regulatory networks in situ.

Early transcriptomic studies using cDNA microarrays, followed by bulk RNA sequencing (RNA-seq) provided foundational insights into transcriptional changes in aging MuSCs, highlighting disruptions in myogenic programs, mitochondrial function, and inflammatory regulation.^7,14,15^ In particular, bulk RNA-seq expanded discovery beyond predefined probes, enabling less biased identification of age-associated transcriptional features, including low-abundance genes and alternative isoforms. Collectively, these studies helped establish aging as a multifaceted process involving both intrinsic transcriptional dysregulation and extrinsic niche perturbation. However, the bulk nature of these techniques averaged gene expression across thousands of cells, thereby obscuring cell-to-cell variability and masking rare or transient subpopulations with distinct functional relevance (Fig. 1). These limitations underscored the need for higher-resolution approaches capable of deconvolving the cellular heterogeneity of the MuSC pool and resolving lineage-specific aging trajectories.

The advent of single-cell RNA sequencing (scRNA-seq) and related multi-omic technologies has transformed this landscape by enabling high-resolution dissection of MuSC states, fate transitions, and interactions with the niche. These approaches have revealed that aging is accompanied by selective loss of quiescent subtypes, emergence of dysfunctional states, and dynamic epigenetic remodeling.^16–18^ Moreover, spatial transcriptomic approaches have begun to preserve tissue context, revealing how aged MuSCs lose positional identity and niche connectivity.^19,20^ Together, these studies suggest that aging affects not just the intrinsic transcriptional state of MuSCs but also their spatial organization and responsiveness to local cues.

In parallel, computational advances — including RNA velocity, multi-omics integration, and machine learning — now enable researchers to predict aging trajectories, identify rejuvenation targets, and simulate stem cell–niche interactions with increasing precision.^21–24^ AI-driven models have even begun to reclassify aging patterns and suggest candidate interventions on the basis of transcriptional and epigenetic drift.^25,26^

In this Perspective, we trace the evolution of transcriptomic and computational technologies that have advanced our understanding of MuSC aging — from early bulk approaches to cutting-edge spatial multi-omics. While these technologies have broad applications across biology in general and skeletal muscle biology in particular, we focus here on MuSCs and how they age, highlighting how transcriptomic resolution has revealed their heterogeneity, fate dynamics, and age-associated decline. We integrate findings across platforms to highlight key themes in transcriptional regulation, fate dynamics, niche influence, and computational modeling.

## Bulk transcriptomics: establishing the foundation

### Early methodologies: cDNA microarrays and bulk RNA-seq

Early studies investigating age-associated transcriptional changes in MuSCs relied primarily on microarray and bulk RNA-seq technologies. Microarray analyses provided some of the first genome-wide views into how aging alters gene expression in MuSCs. These studies revealed that aged MuSCs exhibit reduced expression of genes involved in protein synthesis, mitochondrial function, and metabolism.^14^ Transcriptional profiling also revealed that aged MuSCs acquire intrinsic defects in self-renewal capacity, linked to dysregulated p38α/β mitogen-activated protein kinase (MAPK) signaling and impaired fibroblast growth factor receptor 1 (FGFR1) activity, changes that are not rescued by a young environment.^27^

Other early studies offered insights into transcriptional regulators of MuSC quiescence and activation. Comparative analyses of quiescent and activated MuSCs in vivo identified *Dach1* as a corepressor of cell cycle progression and highlighted markers of quiescence such as *Calcr*, *Cyclin E1*, and *Nap1l5*, along with elevated expression of antioxidant genes.^28^ Genome-wide expression profiling revealed roles for zinc finger proteins and Ephrin signaling, particularly EPHB1, in regulating MuSC self-renewal and myogenic progression.^29^ These studies provided early evidence for transcriptional programs that maintain MuSC function during aging and regeneration.

Regulatory network analysis of quiescent MuSCs identified a tightly controlled set of transcription factors, including Pax7, Myf5, Foxo1, Foxo3, Gli2, and Nfat5, consistent with programs that maintain quiescence.^15^ That study also revealed age-related epigenetic changes, which are addressed in the multi-omics section below.

With the emergence of bulk RNA-seq, it became possible to more comprehensively characterize the transcriptional hallmarks of aging. These included downregulation of myogenic regulatory genes such as *Pax7*, *Myf5*, and *Cyclin D1*, as well as impairments in Notch signaling and oxidative phosphorylation pathways.^6,7,30^ Studies also reported upregulation of inflammatory and senescence-associated genes,^6,7^ further defining the aging MuSC transcriptional landscape.

In vivo transcriptomic profiling using metabolic RNA labeling revealed that quiescent MuSCs maintain low-level transcription of specific gene subsets and undergo rapid transcriptomic shifts during isolation, reflecting early activation and stress responses.^31^ These findings underscored the importance of preserving physiological context during transcriptomic analysis. A related study developed an ex vivo fixation technique to preserve the native transcriptomic state of MuSCs, revealing that conventional isolation methods induce transcriptional and epigenetic artifacts and identifying novel quiescence-associated markers such as *HOXA9*.^32^

Bulk RNA-seq has also been used to study transcriptional dynamics during regeneration and aging. Time-series profiling of injured muscle revealed stage-specific transcriptional waves, ranging from early inflammatory and immune activation to mid-phase myogenic and extracellular matrix (ECM)-remodeling gene expression and late-phase tissue maturation.^33^ These findings illustrate the temporal complexity of MuSC-driven repair and highlight the ability of bulk RNA-seq to capture regeneration dynamics.

Beyond rodent models, transcriptomic profiling of human MuSCs has uncovered therapeutic candidates for reversing age-associated decline. Nutrient screening combined with RNA-seq identified nicotinamide (NAM) and pyridoxine (PN) as synergistic enhancers of MuSC proliferation and differentiation, acting through β-catenin and AKT signaling.^34^ Supplementation with NAM and PN improved muscle regeneration in aged mice and enhanced myogenic potential in human myogenic progenitors.^34^

Population-scale studies have also leveraged bulk RNA-seq to identify transcriptional biomarkers of aging. Analyses of hundreds of publicly available human skeletal muscle transcriptomes revealed conserved dysregulation of calcium handling, PPAR signaling, and neurotransmitter recycling in aging tissue.^21^ Machine learning models trained on these data achieved high accuracy in age prediction, emphasizing the utility of large-scale RNA-seq for biomarker discovery.^21^

Building on these foundations, transcriptomic analyses investigated how circadian and metabolic cues modulate the aging MuSC transcriptome. Aging was shown to disrupt rhythmic expression of genes involved in inflammation, metabolism, autophagy, and mitochondrial function, alterations that were partly reversible through long-term caloric restriction.^35^ Additional studies demonstrated that intrinsic and systemic circadian clocks differentially regulate transcriptional oscillations in MuSCs, particularly for genes related to lipid metabolism and tissue repair.^36,37^ These findings suggest that temporal and systemic regulation significantly influence age-related transcriptional programs and may offer new therapeutic entry points.

Transcriptomic profiling has also identified glutathione metabolism as a key regulator of functional heterogeneity in aged MuSCs, supporting a stress-response model involving nuclear factor erythroid 2–related factor 2 (NRF2) and nuclear factor kappa B (NF-κB) signaling pathways.^38^ Age-associated metabolic reprogramming was also reflected in reduced mitochondrial function and increased reliance on glycolysis.^30^ The observed restoration of *Cyclin D1* expression and rejuvenation of aged MuSCs through exercise further emphasized the utility of bulk RNA-seq for capturing molecular signatures of aging.^7^

While these transcriptomic approaches provided foundational insights into MuSC aging, their reliance on population-averaged signals limited resolution of cellular heterogeneity. As a result, rare subpopulations and dynamic transcriptional states were obscured, prompting a transition toward single-cell technologies to achieve higher-resolution insight into the aging MuSC landscape.

## scRNA-seq: a game changer

### Resolving MuSC heterogeneity and aging trajectories

The advent of scRNA-seq revolutionized the study of MuSCs, revealing age-associated transcriptional dynamics and heterogeneity that bulk methods could not resolve. scRNA-seq of human MuSCs identified two major subpopulations: a quiescent subset enriched for oxidative phosphorylation and a more activated subset marked by ribosomal activity and inflammatory signaling.^16^ Aging skews this balance toward dysfunctional early-activated MuSCs.

Subsequent studies demonstrated that MuSC aging is not purely stochastic. Instead, aging follows reproducible patterns, with selective loss of subpopulations, such as TNFRSF12A^+^ and ICAM1^+^ MuSCs, and expansion of senescence-prone states.^17,39^ RNA velocity analyses further revealed that aged MuSCs exhibit delayed differentiation, with elevated expression of *Snai2* and diminished expression of *Myog* and *Mef2c*.^22^ While RNA velocity provides a powerful framework for inferring dynamic trajectories,^40^ it assumes simplified transcriptional kinetics that may not always hold. To address these limitations, several alternative methods have been developed, including steady-state and dynamical formulations of RNA velocity, as well as deep learning-based models. For instance, Variational Inference for Trajectory by AutoEncoder (VITAE) integrates a latent hierarchical mixture model with variational autoencoders to infer trajectories;^41^ LatentVelo employs probabilistic modeling of splicing kinetics;^42^ and ensemble approaches such as scTEP and CellRank combine pseudo-time ordering with probabilistic state-transition mapping.^43,44^ Together these methods provide complementary strategies for capturing differentiation trajectories in aging MuSCs.

Although originally designed for scRNA-seq, RNA velocity has also been applied to snRNA-seq datasets, where studies demonstrate strong concordance between nucleus- and cell-derived velocity estimates in matched samples;^45^ however, differences in spliced-to-unspliced ratios in nuclear transcripts require cautious interpretation. Complementary human atlas data integrating snRNA-seq and single-cell assay for transposase-accessible chromatin using sequencing (scATAC-seq) further resolved quiescent, early primed, late primed, and differentiating MuSC states, showing that aging skews the balance toward prematurely primed (*FOS*^*+*^, *JUN*^*+*^) MuSCs and providing evidence for progressive exhaustion of the stem cell pool.^18^ In parallel, other studies identified persistent *Notch2*-expressing subsets throughout quiescence and activation, suggesting enhanced self-renewal potential of aged MuSCs.^46^

While these single-cell studies significantly deepen our understanding of MuSC heterogeneity and aging trajectories, they do so in dissociated contexts, devoid of spatial relationships. To fully grasp how stem cells interact with their microenvironment, we turn to emerging in situ technologies.

### Advances in in situ technologies for heterogeneity studies

Spatial transcriptomics and in situ sequencing methods now enable transcriptional profiling within native tissue architecture, revealing positional information, disrupted localization, and altered niche interactions with aging. To date, however, most spatially resolved insight into MuSC aging has come from injury and regeneration paradigms, where activated MuSCs are more abundant and easier to detect. For example, a recent mouse atlas combining scRNA-seq with high-resolution spatial maps of young through geriatric post-injury muscles revealed elevated senescent-like MuSC subsets in aged injury zones, providing direct niche insights beyond fibrotic pathology.^19^ In humans, multi-modal single-nucleus atlases have identified age-related shifts in niche composition,^18^ but these datasets largely lack true spatial localization. Importantly, in both mouse and human, spatial multi-omic maps that robustly localize rare MuSCs in uninjured, physiologically aged muscle remain scarce, reflecting current constraints in sensitivity, resolution, and in situ cell-type discrimination. Consistent with this limitation, spatial analyses have reported loss of spatially defined MuSC niches, alongside increased expression of the senescence markers *Cdkn2a* and *Cdkn1a*, as well as pro-inflammatory cytokines.^19^

Next-generation spatial technologies such as Seq-Scope have enabled submicron-resolution mapping of hybrid muscle fibers expressing mixed transcriptional identities, offering insights into fiber type transitions and age-related cellular reprogramming.^20^ While these fiber-resolving approaches provide valuable architectural context, they have not yet been widely applied to the study of MuSC aging. A systematic spatial transcriptomic atlas of non-fibrotic aging muscle has yet to be reported, in part owing to current limitations in resolution and transcript-detection sensitivity, as well as challenges in distinguishing MuSCs from closely neighboring cell types in situ. However, as spatial resolution and sensitivity improve, these tools will be increasingly capable of capturing the nuanced microenvironments that influence MuSC function during aging. In this context, spatial transcriptomics represents a critical step toward integrating tissue architecture with molecular profiling to better understand stem cell aging dynamics.

## Beyond transcriptomics: Insights from multi-omics integration

### Combining transcriptomics, proteomics, and epigenomics

Integrating transcriptomic data with proteomic and epigenomic layers offers yet another dimension of insight into how MuSCs age, revealing not only molecular changes but also the potential for intervention.

Comprehensive profiling of aged MuSCs revealed a loss of quiescence-associated genes and enrichment of inflammatory, metabolic, and chromatin remodeling pathways.^18^ Transcriptional heterogeneity increases with age, partly owing to stochastic DNA methylation drift at promoter regions.^47^ This drift correlates with reduced transcriptional coordination and increased expression variability, especially for ECM remodeling genes. Epigenetic profiling further demonstrated an age-associated accumulation of repressive H3K27me3 marks and the formation of bivalent chromatin domains, which limit the expression of regenerative genes and contribute to MuSC dysfunction.^15^ Proteomic data revealed age-associated declines in oxidative phosphorylation, mitochondrial translation, and RNA splicing proteins. Restoration of the RNA-binding protein CPEB4 reversed these defects and improved mitochondrial metabolism.^8^ Additional transcriptomic data identified glutathione metabolism and the NRF2/NF-κB axis as key regulators of MuSC heterogeneity and stress adaptation.^38^ Multi-omic profiling further revealed that prostaglandin E2 (PGE2) can reverse aged MuSC dysfunction, leading to enhanced regeneration and muscle strength.^48^ Regulatory shifts were also evident in aging-associated depletion of Psat1 and its metabolic products α-ketoglutarate and glutamine, which are necessary for MuSC activation and proliferation.^49^ Bulk and single-nucleus RNA-seq analyses of muscle biopsies across the lifespan identified reduced MuSC abundance with age and downregulation of ECM-related genes. Upregulation of mitochondrial stress response pathways, as well as the emergence of regenerative MuSC subclusters expressing *LGR5* and *MYH7B*, point to transcriptional heterogeneity and residual regenerative potential.^50^ Joint scRNA-seq and scATAC-seq mapping further captured dynamic changes in gene regulatory networks and chromatin accessibility during aging.^51^

### Environmental signals and niche interactions beyond the transcriptome

The microenvironment plays a crucial role in preserving MuSC identity and quiescence. Aging disrupts this niche support, driving a transition toward a pro-inflammatory transcriptional state.^35,52^ Extrinsic signals from fibro-adipogenic progenitors and macrophages also modulate MuSC activation, proliferation, and differentiation during both regeneration and aging.^53–55^ These niche changes include increased expression of inflammatory cytokines such as CXCL14 and cell cycle inhibitors such as CDKN1C and CDKN2A, coupled with downregulation of ECM and adhesion genes, including *CAV1*, *SPRY1*, *FN1*, and *ITGB1*.^56^

Seminal heterochronic parabiosis experiments revealed that systemic factors from young animals can rejuvenate the aged MuSC niche and restore regenerative function,^57,58^ emphasizing the niche’s plasticity and its impact on MuSC function. Likewise, transplantation of aged MuSCs into a young niche can reverse many of these changes, restoring youthful gene expression profiles and chromatin accessibility.^17,59^ Re-quiescence defects in aged MuSCs were further linked to reduced expression of perlecan (Hspg2), a myotube-derived ECM component necessary for quiescence re-entry.^60^ Supplementation with endorepellin, a perlecan fragment, restored quiescence and reduced fibrosis in aged muscle.

These findings demonstrate that aging is not a unilateral loss of stemness but rather a rewiring of stem cell–niche interactions. Understanding how niche degeneration biases MuSC state transitions is crucial for designing rejuvenation strategies.

### Plasticity and rejuvenation potential revealed by multi-omics

Although aged MuSCs show pervasive decline, emerging evidence highlights their surprising plasticity and the possibility of functional rescue. Functional impairment in aged MuSCs is accompanied by a shift in transcriptional programs, with increased expression of activation markers such as *FOS*, *JUN*, and *EGR1* and reduced expression of quiescence-associated genes including *CALCR* and *FOXO3*.^18^ However, this state retains plasticity: voluntary exercise has been shown to partially restore youthful transcriptional signatures, particularly in MuSC subsets involved in glycolysis and mitochondrial function.^52^

A subset of MuSCs associated with the neuromuscular junction (NMJ) also adopts maladaptive, synaptic-like gene expression programs during aging or denervation.^61^ Restoration of NMJ integrity was able to normalize these states and improve regenerative potential, suggesting that localized environmental signals can modulate MuSC aging phenotypes.

While these molecular shifts illustrate MuSC decline, they also reveal unexpected windows of plasticity. Understanding how niche interactions and environmental signals modulate this potential is therefore critical for developing rejuvenative strategies.

## Emerging computational tools

**Fig. 2:**
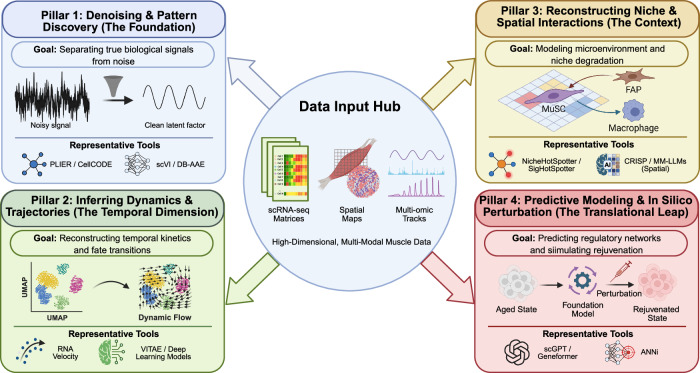
Computational framework for modeling aging and rejuvenation dynamics in MuSCs. High-dimensional, multi-modal muscle datasets serve as the central input for four key analytical pillars designed to resolve biological complexity. Pillar 1 (Denoising): removes technical noise to reveal true biological signals (e.g., using scVI). Pillar 2 (Dynamics): reconstructs cellular timelines to map cell fate transitions and aging trajectories (e.g., RNA velocity). Pillar 3 (Spatial context): models the stem cell niche and interactions with neighboring cells (e.g., NicheHotSpotter). Pillar 4 (Prediction): uses AI to predict cellular responses to potential rejuvenation treatments (e.g., scGPT).

As single-cell and single-nucleus omics technologies uncover the cellular heterogeneity of MuSC adaptation to aging, the integration of artificial intelligence (AI) and machine learning (ML) has become increasingly important. These computational approaches support trajectory inference, prediction of cell state transitions, and reconstruction of niche interactions from high-dimensional datasets (Fig. 2). This paradigm shift has been emphasized in recent commentaries on muscle plasticity research.^62^

### From statistical models to ML: foundations in bulk and single-cell analysis

Early statistical methods such as CellCODE introduced latent variable models for deconvoluting cell-type-specific signals from heterogeneous bulk RNA-seq data without requiring explicit cell proportion measurements.^63^ Building on this approach, Pathway-Level Information Extractor (PLIER) further integrates biological priors, such as pathway and marker gene sets, to improve interpretability and separate biological variation from technical noise — particularly valuable for aging muscle tissue, in which subtle transcriptomic shifts are challenging to resolve.^64^

More sophisticated ML and AI approaches are now transforming the analysis of single-cell and multi-omics datasets. These tools uncover hidden patterns, predict cellular behaviors, and identify potential therapeutic targets in MuSC aging. One early application involved training five ML models on 545 publicly available skeletal muscle transcriptomes to predict donor age, with the Deep Feature Selection model achieving a mean absolute error of 6.24 years, followed closely by a Support Vector Machine model.^21^ This analysis also revealed dysregulated pathways in aged muscle, such as calcium homeostasis, PPAR signaling, and neurotransmitter recycling, highlighting potential biomarkers of muscle aging.

### Transformer-based models and foundation architectures

More recent applications of artificial neural networks (ANNs) have moved beyond classification to infer regulatory interactions. The Applying ANN inference (ANNi) model, trained on human muscle transcriptomes, uncovered aging regulators including CHAD, ZDBF2, USP54, and JAK2, as well as exercise-responsive genes such as *SCFD1*, *KDM5D*, *EIF4A2*, and *NIPAL3*, modeling directed regulatory interactions that prioritize candidate intervention targets while recognizing that definitive causality requires validation through orthogonal perturbation.^24^ Transformer-based models such as Geneformer and scGPT represent the next generation of AI tools. These foundation models are pretrained on massive single-cell and multi-modal datasets, enabling high-resolution tasks such as cell type annotation, regulatory network reconstruction, and simulation of perturbations.^26,65–67^ Notably, scGPT employs a generative pretraining strategy using over 10 million single-cell profiles, supporting zero-shot transfer learning and predictive modeling of gene expression under hypothetical perturbations — an approach with significant implications for modeling MuSC aging trajectories and computational rejuvenation.^67^ Although scGPT has not yet been applied directly to MuSC datasets, its architecture illustrates how foundation models can be repurposed to address tissue-specific questions, including those in skeletal muscle. By contrast, scVI, a variational autoencoder framework widely used in single-cell analysis, has already been applied to datasets containing MuSCs, such as those from the Tabula Sapiens Project,^68,69^ and serves as a useful benchmark for modeling single-cell variation.

### Task-specific tools for muscle and stem cell aging

Several task-specific tools have also emerged for studies of aging muscle and stem cells. NicheHotSpotter, a general framework for cell-niche inference that integrates signaling interactomes with gene regulatory networks, was applied to skeletal muscle to prioritize insulin-like growth factor-1 (IGF-1)/Akt-centered nodes linked to age-associated MuSC decline.^25^ Similarly, SigHotSpotter uses a probabilistic Markov chain to infer signaling “hotspots” from scRNA-seq data, identifying key regulators that stabilize stem cell phenotypes across various tissues, including muscle.^70^

CRISP, a deep-learning-based spatial image analysis tool, enables high-resolution proteomic profiling and cell-type deconvolution, expanding our understanding of tissue-level aging dynamics.^71^ The DeepNEU platform,^72^ originally developed to model induced pluripotent stem cell differentiation and regenerative potential, illustrates how unsupervised frameworks can be conceptually extended to MuSC research. In hematopoietic systems, ANNs have revealed age-related shifts in stem cell division modes,^73^ offering mechanistic concepts that may be transferable to MuSC biology. Together, these tools illustrate the potential for repurposing general deep learning models for MuSC-specific aging research.

Improving data quality remains another frontier. The Dynamic Batching Adversarial Autoencoder (DB-AAE) addresses common single-cell challenges such as dropout noise, enhancement of pseudo-time inference, and rare cell detection.^23^ While DB-AAE itself is a general algorithm, its application to muscle datasets demonstrates how such frameworks can refine resolution in MuSC studies, despite not being designed specifically for this tissue.^23^

### Advanced AI frameworks for therapeutic discovery and spatial modeling

AI is also accelerating therapeutic discovery. A high-throughput ML-driven screen identified 3-deazaadenosine as a molecule that rejuvenates aged MuSCs by restoring epigenetic marks and proliferation capacity.^74^

As spatial transcriptomics and multi-modal data become central to understanding aging tissues, new AI frameworks are emerging to integrate these layers. Multi-modal large language models (MM-LLMs) are being explored for their ability to integrate spatial transcriptomics, histology, and clinical metadata. For instance, MM-LLMs could support reconstruction of 3D spatial multi-omics atlases, helping to model age-related niche remodeling and simulate high-resolution therapeutic interventions in MuSCs.^75^

Together, these computational advances are redefining the landscape of MuSC aging research. In particular, they provide a framework for predicting erosion of stemness subtypes, integrating niche-derived cues with intrinsic aging programs, and simulating how rejuvenation strategies may reshape MuSC trajectories. At the same time, most AI approaches remain constrained by training data bias, limited interpretability, and simplifying assumptions about gene regulatory relationships, underscoring the need for careful benchmarking against experimental evidence. As datasets grow increasingly high-dimensional and multi-modal, AI and ML tools will be indispensable for converting complex biological signals into actionable regenerative strategies.

## Conceptual models of MuSC pool remodeling with age: From stochastic drift to functional erosion

**Fig. 3:**
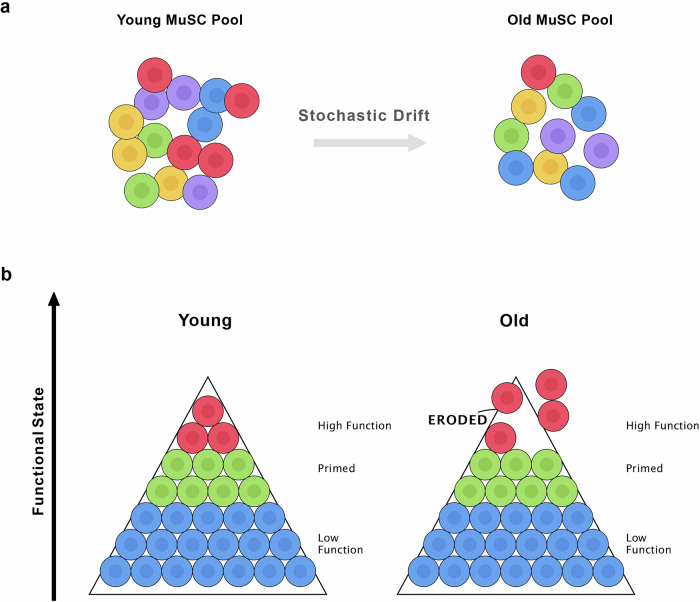
Two conceptual frameworks for remodeling of the MuSC pool with age. This figure illustrates two intersecting models describing how the MuSC pool changes with age. **a** Stochastic drift model. Conceptual depiction of MuSC lineage behavior inferred from random lineage-labeling or barcoding, in which colors represent initially randomly labeled lineage units. Over time, labeled lineages can expand or contract, reflecting stochastic drift in lineage size and occasional attrition (loss or dysfunction). In the context of aging, the MuSC pool can decline through probabilistic loss or dysfunction of MuSCs without necessarily collapsing overall state diversity or heterogeneity. **b** Pseudo-hierarchical erosion model. MuSCs can be organized into functional tiers, ranging from high-function “reserve” states (e.g., Pax7^High^, Myf5^Low^, GSH^High^, and CD34^High^) at the apex, to intermediate primed states, and low-function committed progenitors at the base. Aging leads to targeted depletion of the top functional tiers, eroding the hierarchy from the peak downward. This structured loss results in impaired regenerative capacity despite maintenance of overall pool size.

A central question in MuSC aging is how cell fate decisions are regulated, maintained, and ultimately disrupted over time. Early models of MuSC behavior emphasized either a stochastic framework, in which fate decisions occur randomly and reversibly at the population level, or a hierarchical framework, in which functionally distinct subsets are progressively lost. Increasing transcriptomic resolution, together with functional studies, suggests that MuSC aging is best explained by integrating both views. Here, we outline two intersecting conceptual frameworks that describe how the MuSC pool changes with age, emphasizing how these models emerge from lineage- and transcriptome-based evidence (Fig. 3).

### Stochastic drift: preserving state diversity despite depletion

Lineage-labeling and barcoding studies support a population-asymmetry framework in which MuSC fate decisions are probabilistic and reversible at the population level, rather than dictated by fixed sublineages.^76^ In these paradigms, initially random lineage labels can expand or contract over time, consistent with stochastic drift in lineage size and occasional attrition. Although most of these data were generated in regenerative settings rather than during steady-state aging, they support a model in which aging can reduce the MuSC pool through probabilistic loss or dysfunction of MuSCs (or labeled lineages) without implying deterministic, subset-specific collapse of heterogeneity (Fig. 3a).

Single-cell barcoding and dynamical systems approaches further support this view, showing that aged MuSCs follow the same differentiation trajectories as young cells but stall near commitment checkpoints, suggesting delayed progression rather than lineage restriction.^22^ These findings reinforce the idea that aging alters the kinetics of fate transitions rather than enforcing rigid lineage bifurcations, consistent with a model of dynamic, non-hierarchical regulation.

### Pseudo-hierarchical erosion: Selective loss of high-function “reserve” states

In contrast to a purely stochastic view, recent transcriptomic and functional analyses have identified reproducible subpopulations of MuSCs with enhanced regenerative potential — such as Pax7^High^, MYF5^Low^, GSH^High^, and CD34^High^ states — that are disproportionately lost with age.^38,77–79^ Conceptually, these states can be organized into functional tiers, with highly functional “reserve” states at the apex, intermediate primed states in the middle, and committed progenitors at the base (Fig. 3b).

Consistent with this framework, MYF5^Low^ MuSCs associated with long-term self-renewal decline with age, whereas MYF5^High^ cells dominate the aged MuSC pool.^78^ Adding to this hierarchical perspective, scRNA-seq profiling further revealed distinct transcriptional states between Pax7^High^ and Pax7^Low^ MuSC subpopulations.^77^ Pax7^High^ cells are enriched for stemness-related genes and are preferentially associated with glycolytic myofibers, whereas Pax7^Low^ cells express markers of myogenic differentiation.^77^ Likewise, MuSCs with higher levels of CD34 expression exhibit reduced differentiation propensity and are preferentially depleted with aging.^79^ The selective depletion of these high-function states argues against a uniform decline and supports a pseudo-hierarchical erosion model, in which aging preferentially erodes the most potent functional tier, shifting the pool composition toward primed and committed states.

Importantly, we use the term “pseudo-hierarchical” to emphasize that these tiers may not represent fixed lineages; it remains unclear whether these dichotomies reflect distinct hierarchies or overlapping axes of cell fate. A key caveat is that subpopulations defined solely by gene expression levels — such as *Pax7* or *CD34* — may reflect dynamic states rather than stable cellular identities. For instance, there is no definitive evidence that CD34^High^ and CD34^Low^ cells represent fixed subtypes rather than a fluid population undergoing temporal shifts in expression. This limitation applies broadly to single-time-point analyses and highlights the need for more integrative, longitudinal approaches to determine whether observed transcriptional states represent stable hierarchies or transient phases within a dynamic continuum. Understanding how these molecular states intersect with stochastic behavior will be key to resolving how MuSC heterogeneity is maintained and altered during aging.

Together, stochastic drift and pseudo-hierarchical erosion provide a unified framework for interpreting MuSC aging as changes in both pool size and state composition. A central implication is that loss of MuSCs with age does not necessarily require a collapse of state diversity; aging can reduce regenerative output by altering which states persist and how efficiently cells transition between states. Moving forward, longitudinal single-cell and single-nucleus profiling — ideally paired with in situ approaches and functional readouts — should enable quantitative tests of how state occupancy and state-transition kinetics jointly predict regenerative competence and whether interventions that stabilize or restore “reserve-like” states can meaningfully sustain regeneration during aging.

## Conclusion

The past two decades have witnessed a paradigm shift in the study of MuSC aging, fueled by continuous advances in transcriptomic technologies and the analytical frameworks built around them. Early microarray studies provided the first genome-wide snapshots of age-associated changes in gene expression, whereas the adoption of bulk RNA-seq enabled more comprehensive and less assumption-driven discovery, including detection of low-abundance transcripts and isoform-level regulation. These population-level approaches established foundational transcriptional signatures of aging MuSCs, but they averaged across cells and obscured heterogeneity. The emergence of single-cell and single-nucleus transcriptomics revealed previously hidden heterogeneity and enabled trajectory-level analysis of MuSC fate dynamics. These datasets demonstrated that MuSC aging is not purely stochastic, revealing reproducible shifts in state composition, altered kinetics along myogenic trajectories, and selective vulnerability of specific functional states. Spatial transcriptomic approaches, while still constrained by sensitivity and cell-type discrimination in muscle, are beginning to place these MuSC states back into the context of local tissue neighborhoods, strengthening links between transcriptional states and niche organization during aging and regeneration. In parallel, multi-omic strategies that incorporate chromatin accessibility or metabolic readouts have provided additional mechanistic support by connecting age-associated transcriptional programs to epigenetic remodeling and metabolic state changes.

Perhaps most critically, these increasingly high-dimensional transcriptomic datasets have empowered a new generation of computational tools that not only model and predict aging trajectories but also simulate interventions capable of reversing them. Trajectory inference, dynamical modeling, and machine learning are now routinely used to predict aging-associated state shifts, prioritize perturbation targets, and generate testable hypotheses for rejuvenation strategies. Together, these advances suggest that MuSC aging is neither purely stochastic nor immutable; rather, it arises from a complex interplay of regulated transcriptional programs, epigenetic states, and environmental context, many of which remain modifiable.

Conceptually, the transcriptomic era has clarified that two intersecting frameworks are useful for interpreting MuSC aging. Stochastic drift captures how population-level state diversity can be retained even as the pool is reduced through probabilistic drift and attrition. Pseudo-hierarchical erosion captures the structured depletion or destabilization of high-function reserve states — defined by multi-dimensional stem cell fitness, including quiescence stability, self-renewal capacity, stress resilience, and activation competence — and the downstream consequences for regenerative potential. Importantly, many transcriptomically defined MuSC states likely represent positions along a dynamic continuum rather than fixed states, emphasizing the need for longitudinal measurements and functional validation.

Future progress will depend on integrating multimodal, longitudinal datasets with AI-driven models capable of generating testable hypotheses about MuSC–niche interactions, stemness erosion, and rejuvenation strategies, ultimately bridging basic discovery with clinical translation. Looking forward, the integration of complementary technologies — bulk, single-cell, spatial, and multi-omics — together with computational modeling will be critical for connecting molecular-level discoveries in MuSC biology to systemic physiology and, eventually, to translational interventions for muscle aging.
